# Convergent Validity of the Patient Reported Outcome Measurement Information System-Pediatric Physical Activity Instrument (PROMIS^®^-PA) with Wearable Devices in Adolescents

**DOI:** 10.3390/children10060940

**Published:** 2023-05-26

**Authors:** Reem A. Algheryafi, Katherine B. Bevans, Shivayogi V. Hiremath, Jin-Shei Lai, Carole A. Tucker

**Affiliations:** 1 Department of Physical Therapy, Mohammed Al-Mana College for Medical Sciences, Dammam 34222, Saudi Arabia; 2 Patient Reported Outcomes, Janssen Global Services LLC, Horsham, PA 19044, USA; kbevans@its.jnj.com; 3 Department of Health & Rehabilitation Sciences, Temple University, Philadelphia, PA 19122, USA; 4 Department of Medical Social Sciences, Northwestern University, Chicago, IL 60611, USA; js-lai@northwestern.edu; 5 Nutrition, Metabolic & Rehabilitation Sciences, School of Health Professions, University of Texas Medical Branch, Galveston, TX 77555, USA

**Keywords:** physical activity, adolescents, wearables, self-reported outcome measure, validity, step counts, PROMIS, ecological momentary assessment

## Abstract

The study was conducted mainly to examine the convergent validity of the Patient Reported Outcome Measurement Information System-Pediatric Physical Activity instrument (PROMIS^®®®^-PA) with step counts from wearable devices and another validated self-reported outcome measure. As a secondary aim, we explored the effect of different recall time frames (7-day, end-of-day [EoD], and ecological momentary assessment [EMA] time frames during the day) in terms of their feasibility and associations with each other and with step counts. This was a prospective cohort study that examined the associations between measures of PA in school-age children and adolescents (*n* = 84, aged 10–20). The participants wore Fitbit devices for 7 consecutive days, and then completed the 7-day-recall PROMIS-PA short form and Youth Activity Profile (YAP). Additional analyses were completed in a sub-sample (*n* = 25, aged 11–18 years) using the PROMIS-PA for the EMA at five intervals during the day (shorter form) and at the EoD. In the total sample, the PROMIS-PA results showed positive moderate correlations with the YAP and average daily steps (*r* = 0.533, *p* < 0.001 and *r* = 0.346, *p* = 0.002, respectively). In the sub-sample, the 7-day PROMIS-PA was highly correlated with the averaged EMA or EoD ratings for the week, and moderately correlated with the daily step counts. These findings support the validity of the PROMIS-PA as a measure of self-reported physical activity. Adolescents demonstrated higher compliance rates and preference for the 7-day recall and EoD assessments compared to more frequent EMA reporting.

## 1. Introduction

The positive effects of physical activity (PA) on physical and mental health and the importance of maintaining regular adequate levels of PA are well known [1,2]. Decreased PA is common during adolescence, and this decline often continues to adulthood [3,4]. To better capture the effectiveness of PA intervention strategies in this age group, psychometrically sound instruments to assess PA in school-age children and adolescents are critical.

PA is commonly assessed using wearable devices to record motion of the body, and/or with self-report questionnaires [5,6]. Some of the challenges encountered with using wearable devices include a lack of consensus in the methodology used to collect and analyze the data, missing data due to participant non-adherence, cost, and inadequacy in capturing some types of PA performed with restricted motion [7,8]. Measuring PA with self-report instruments is considered to be more practical and economical in epidemiological studies, and these instruments can provide relevant contextual information as well [5,9]. Conducting rigorous studies to establish measurement psychometric properties of self-reported PA in school-age children and adolescents is essential to ensure the valid assessment of PA [10].

The Patient Reported Outcome Measurement Information System (PROMIS)^®^, supported by the National Institutes of Health (NIH), developed psychometrically robust self-reported outcome measures across multiple health domains for use as clinical trial endpoints [11,12]. The Pediatric PROMIS Physical Activity (PROMIS-PA) measure was developed using a mixed-methods approach [13,14,15]. However, the association of PROMIS-PA scores with wearable devices has only been reported in a small sample of children with cancer [16], but, to our knowledge, not in typically developing children. The convergent validity of the PROMIS-PA with the self-reported Youth Activity Profile (YAP) [17] was only reported for youth attending a community event, with both measures using a single 7-day recall [18]. The researchers in the previous study examined the convergent validity of the longer version of the PROMIS-PA [18]. Therefore, the convergent validity of the PROMIS-PA (8-item short form) with an objective PA monitoring using wearable devices as well as another self-reported instrument needs to be investigated.

The PROMIS-PA instrument uses a 7-day recall period, which requires recall over an extended period, and may contain recall bias [19]. The ecological momentary assessment (EMA), which involves more frequent reporting (e.g., several times a day or daily) of a behavior of interest (e.g., nutrition, PA, pain, affective constructs, fatigue, etc.), has emerged as an option for self-reporting to reduce recall bias [19,20,21,22,23]. The common key features of the EMA are as follows: (1) it allows respondents to report behaviors occurring in their natural environment; (2) the reporting is based on current or very recent feelings or behaviors by respondents, rather than relying on a long-period recall or summary; (3) the timings selected for assessment are event-based or time-based (following time schedule intervals); and (4) it captures more information about variations in behavior due to the multiple assessment responses collected over time [20,21]. As an example, PA was assessed in some studies using the EMA reporting method by asking participants about the performed PA (e.g., type of current performed activity) at specific times (right now, right before the beep, etc.) when the question was delivered to them through mobile phones [20,23]. The frequency and timing of the EMA in application varies; less frequent EMAs (1–2 times a day such as the end-of-day reporting [EoD]) may lower the response burden and be sufficient to reduce recall bias [20]; however, frequent EMAs may reinforce the behavior under study.

Currently, the reference objective measure of PA in real-world, community-based applications are wearable devices which measure motion that are commonly expressed as step counts. Since PA is defined as bodily movement, motion sensors in these wearable devices are used to detect motion by recording acceleration signals of the body to estimate PA [6]. In our study, the daily step counts detected by wrist-worn accelerometry-based devices connected to software applications on participants’ electronic devices were collected. Given the considerable challenges in incorporating wearable devices in large population-based studies, improved understanding of the convergent validity of the PROMIS-PA with step counts from wearable devices is needed. Given the varied pros and cons of the various approaches in evaluating PA, understanding the relationship among these methods by comparing data from wearable devices (objective, but wear time and interpretation of step counts are issues), self-reported with 7-day recall (recall bias, but low administrative and user burden is an advantage), and self-reported using the EMA (less recall bias but the greater administrative and user burden may alter behavior under study, given frequent surveys), can benefit researchers and clinicians in their choice of PA measurement mode. This study provides researchers and clinical pediatric providers with information about the convergent validity of the PROMIS-PA 8-item short form with wearable device step counts in typically developing children. The study also highlights feasibility aspects about the administration of the EMA and EoD modes of the PROMIS-PA in adolescents that can be used for designing future studies.

We hypothesized the following: (1) a high positive association between the 7-day-recall PROMIS-PA and the YAP scores; (2) moderate positive associations between the PROMIS-PA, YAP scores, and weekly step counts from wearable devices. In addition, as an exploratory aim, we compared the effect of three different recall modes (7-day recall, EoD, and 5-times daily EMAs) against step counts in a sub-sample of participants.

## 2. Materials and Methods

A prospective, observational study design was used to evaluate the convergent validity of the PROMIS-PA instrument in adolescents.

### 2.1. Participants

We recruited a convenience sample of 84 adolescents aged 10–20 years from the Philadelphia, Pennsylvania, USA region, through personal contacts and using the snowball sampling technique. Participants were included in the study if they were aged 10 to 20 years; spoke and read English; were typically developing with no self/parent reported childhood conditions that could impair their ability to perform age-appropriate movement skills or participate in PA; and had access to a smart phone or tablet for 7 consecutive days. The exclusion criteria were having an intellectual disability that could hinder completion of the questionnaires, an injury or surgical procedure in the past six months with a persistent effect on their level of PA, or a chronic health condition restricting PA. In compliance with the study protocol approved by the Institutional Review Board of Temple University, electronic parental consent and informed child assent were obtained before participation for participants were under 18 years, and participant consent was obtained for adolescents 18 years of age or older. The EMA and EoD reporting data were collected and analyzed for a sub-sample consisting of the first 25 enrolled participants (Table 1). Given the impact and timing of COVID-19-related restrictions on school attendance, this sub-sample was chosen, as all participants were in similar schooling modes (e.g., home-schooled), and the number of participants (*n* = 25) provided adequate statistical power.

### 2.2. Procedures and Measurements

All survey data were collected electronically using the RKStudio^TM^ platform (CareEvolution, LLC). After providing the appropriate consent and/or assent, participants and their parents were provided with a quick start guide to download the Fitbit and MyDataHelps^TM^ applications on their own smart phone or tablet device. MyDataHelps^TM^ is the application through which participants completed all the surveys and shared their Fitbit data, which were synced to the Fitbit application to be uploaded to the RKStudio^TM^ platform.

All participants completed a general health information and sociodemographic questionnaire. As this study was conducted during the COVID-19 pandemic, participants also completed a 3-question survey to report changes in the amount, type, and location of PA compared to before the pandemic. Participants wore Fitbit devices on their wrists, which were connected wirelessly to the Fitbit application, continuously for 7 consecutive days. They received 5 EMA surveys and an EoD survey every day during these scheduled days, and completed the 7-day-recall PROMIS-PA and YAP on the 7th day (Table 2). The given value for each selected option, as explained in the sections below, was considered for calculating a total score for each self-reported instrument used in this study. Each evening, the participants reported whether the Fitbit was taken off during the day (e.g., two participating athletes were required to remove devices for sport participation for only an hour on two days); if so, the time, duration, and reason for not wearing the device was given.

#### 2.2.1. The 7-Day-Recall PROMIS-PA

The 8-item form was used to measure PA lived experiences, with items focusing on the 7-day recall of physiological responses and symptoms to PA (breathed hard, sweated, muscles burned…etc.) as well as short bouts of higher-intensity PA (Appendix A) [15,18]. Each item was scored with a value ranging from 1 to 5, where 1 indicates “no day” and 5 indicates “6–7 days.” The summed scores of these values were used for the purpose of the analysis in this study, with a given total score out of 40 [12]. The instrument development process and psychometric properties have been reported in other studies [13,14,15,18].

#### 2.2.2. EMA and EoD of PROMIS-PA

The 4-item PROMIS-PA form was used with some modifications to the question structures and response options, in order to be appropriate for EMA time periods (Appendix B). These EMA time frames were chosen to be consistent with the 5 specific time periods specified in the YAP to support comparisons across measures. The selection of the time periods was also supported by the participants’ preferences for responding to surveys in our prior qualitative study [24]. The EMA survey time frames were early morning, 6 to 8 a.m.; mid to late morning, 8 a.m. to 12 p.m.; early afternoon, 12 to 3 p.m.; late afternoon, 3 to 6 p.m.; and evening, 6 p.m. until your bedtime (Table A1 in Appendix C). The EMA surveys were scheduled to be delivered to participants on the MyDataHelps^TM^ application by the end of each period, and the last one was available for completion at 8 p.m. The participants were provided with a timetable for survey completion times, and were instructed to complete the surveys as close to the scheduled time frame as allowed (e.g., school schedules). All responses were time stamped and considered to be valid for subsequent analysis if completed within the allocated time segments before the start of the next EMA (e.g., the early morning EMA asking about PA from 6 to 8 a.m. had to be completed before obtaining the next EMA that started at 12 p.m.), and for the 5th daily EMA if completed before 2 a.m. The EMA data were accepted for each day’s analysis if the participants responded to at least 3 of the 5 daily EMA prompts within their allocated time segment during the day [22]. The participants received a daily push notification through the application every morning as a reminder to complete the surveys, and to wear and synchronize their Fitbit. Other push notifications were sent to remind participants to respond if a participant had not responded near the end of the scheduled period.

The 8-item PROMIS-PA short form was used to assess EoD recall of PA with similar modifications to the recall period, with EoD items reframed as “today”. The EoD PROMIS-PA was available on the application for completion every evening at 8 p.m. local time. Responses to the EoD surveys were valid for inclusion in the analysis if the survey was completed before 2 a.m. The response options for the EoD and EMA items were yes and no, which were scored as 1 and 0 values, respectively, and the summed scores for all of the items together in each survey were used for the analysis (the total score was calculated out of 4 for each completed EMA, and out of 8 for each completed EoD survey). If the valid daily data for EoD or EMA (at least 3 EMA reports/day) reporting were available from at least 3 days, the scores from only the non-missing days and non-missing EMAs were averaged for the 7-day analysis [22].

#### 2.2.3. Youth Activity Profile (YAP)

The YAP is a 15-item self-report instrument that was developed to assess PA and sedentary behaviors of youth over the past 7 days [17]. The instrument is divided into activity at school, activity outside of school, and sedentary behaviors sections. The YAP validity was tested in different samples, and the correlations between the YAP and ActiGraph data were 0.38 and 0.52 for school activity and out-of-school activity, respectively [17]. The test-retest reliability examined within 2 to 3 weeks was 0.74 [25]. Since our study aimed to examine the validity of a PA instrument and school activity was impacted by COVID-19 restrictions, we used the modified YAP (removed 5 items related school day activities) to assess activity throughout the day, as in the previous validation study of the PROMIS-PA [18]. The modified YAP included 7 items. The response options for the first 5 items (from “a” to “f”) were scored on a 0–5 scale, whereas the response options (from “a” to “e”) for the last 2 items, asking about PA during the weekend days, were scored on a 0–4 scale. All the values of the selected response options were summed up for each participant to be included in the analysis as a total score out of 33 (Appendix D).

#### 2.2.4. Wearable Devices

Participants were provided with a Fitbit Charge 3 or 4 for the duration of the study, and were instructed to wear it on their non-dominant wrist. Participants were allowed to use their own Fitbit if it had comparable features to the Fitbit Charge. Evidence supports using the consumer-based activity trackers for estimating free-living PA among adolescents [8,26,27]. These accelerometry-based wearable devices, which track step counts based on body motion, are currently considered as the gold standard of PA in real-world, community-based applications. The daily step count data that were included in our analysis were downloaded from the RKStudio^TM^ platform by linking a participant’s Fitbit account to his/her MyDataHelps^TM^ account. The weekly step count was calculated by averaging the daily steps across the total number of days that a participant wore a Fitbit. Consistent with prior reports [26,28,29], a minimum of 3 days of at least 8 h of wear time per day, and with a daily step count over 1500 steps, was required for data to be included in the analysis.

### 2.3. Data Analysis

Descriptive statistics were computed to summarize the participants’ sociodemographic data, survey compliance rate, and PA measurements using mean ± standard deviation and range for continuous variables and frequency and percentage for categorical variables. Parametric statistical analyses were used, since the PA data showed a normal distribution. All statistical procedures were performed using SPSS software, version 27.0 (IBM Corporation), and the significance level was set at 0.05. The Pearson product-moment correlation coefficient was used to examine all the correlations stated in the hypotheses for the total sample and sub-sample. Independent *t* tests were used to examine whether weight, height, 7-day-recall PROMIS-PA scores, YAP scores, and average of daily steps differed between the sub-sample and the rest of sample.

For the data included in the analyses, all 84 participants (the original sample) completed the 7-day recall surveys. Valid step count data, as defined above, were available for *n* = 81 participants (*n* = 3 were lost due to device issues). The daily step count was excluded from the analysis if it was less than 1500 steps. This occurred for a single day for 6 participants and for 3 days for only one participant of the total sample. After the enrollment of the first 25 participants, we noted that the school attendance mode varied highly (i.e., in-person attendance or half-days in-person and half-days online as an impact of COVID-19 related restrictions) for the subsequently recruited participants who tended to complete the EMA or EoD surveys after the scheduled time, or did not complete them at all; these scenarios resulted in having invalid data for inclusion in the analysis. In the sub-sample of *n* = 25, 616 EMA surveys (70.4% of 875 EMA surveys delivered across all days and participants) were completed within the allocated time segments, and 152 of the EoD surveys (86.9% of the 175 EoD surveys delivered) were valid for being completed before 2 a.m. The participants provided an average of 24.64 (SD = 8.35) valid EMA responses out of the 35 delivered EMA surveys. There was no discernible pattern of missing responses across the time segments. The compliance rates for providing valid EMA and EoD responses by days are detailed in Table 3. For the 7-day analyses in the sub-sample of *n* = 25, valid EMA data were available from 21 (84%) participants, and EoD data from 24 (96%) participants.

## 3. Results

In the total sample, the 7-day-recall PROMIS-PA scores had significant positive moderate correlations with the YAP scores and the average of daily steps (*r* = 0.533, *p* < 0.001 and *r* = 0.346, *p* = 0.002, respectively) (Hypothesis 1 and 2). The YAP correlated in a low to moderate manner with the average of daily steps (*r* = 0.327, *p* = 0.003) (Hypothesis 2). In the sub-sample, the 7-day-recall PROMIS-PA scores demonstrated high positive correlations with the averaged EMA and EoD ratings for the week (*r* = 0.748, *p* < 0.001 and *r* = 0.914, *p* < 0.001, respectively) (Table 4). The association of the average of daily steps with the averaged EMA and EoD ratings for the week were comparable to its association with the 7-day-recall PROMIS-PA scores.

Single-day analyses for all days revealed moderately high positive correlations between the EoD scores and average daily EMA scores (*r* range = 0.638–0.945, *p* ≤ 0.004) (Table 5). The average daily EMA scores and daily steps had significant, moderately high positive correlations on all days, except for day 5, which showed a moderate correlation (non-significant). The EoD scores were significantly and moderately to highly associated with the daily steps, except for day 2. The *t* tests showed that weight, height, 7-Day recall PROMIS-PA scores, YAP scores, and average of daily steps did not differ significantly between the sub-sample and the rest of participants in the total sample.

## 4. Discussion

This study assessed the convergent validity of the 7-day-recall PROMIS-PA instrument with other common PA assessment tools in adolescents. The validity of the PROMIS-PA instrument (the 8-item short form) was supported by our study findings, which revealed significant positive associations between the PROMIS-PA (7-day-recall) and YAP scores and the average of daily steps for 7 days measured by wearable devices (i.e., Fitbits). The associations were further explored with the EMA and EoD recall versions of the PROMIS-PA instrument. Previous studies that applied EMA techniques to reduce possible recall bias in self-reporting over similarly long recall periods demonstrated that the EMA is an accurate self-reporting technique for PA when compared with wearable device-derived measures [23,30]. Broderick et al. [22] indicated that EoD reporting is a highly accurate strategy that can replace the EMA when the target is the average of a performed behavior over a period. A study by Knell et al. [31] supported the validity of daily reporting, which demonstrated a better estimate of PA than using the 7-day recall for self-reporting. Both EMA and EoD reporting using PROMIS-PA-based items were employed in this study, and provided evidence that supported the 7-day-recall PROMIS-PA’s representation of PA.

The correlation of the PROMIS-PA with the YAP scores in our sample was comparable to the earlier values in a previous study that used a larger sample (*r* = 0.60, *n* = 348) [18]. This correlation highlights that while both the YAP and PROMIS-PA are measuring PA, the underlying PA constructs are not identical. The PROMIS-PA items focus on the lived experiences of physiological responses to PA and short bouts of higher intensity of activity [15,18], whereas the YAP relies on quantifying the activity level through reporting the time spent being physically active during different periods of time [32]. The association of the 7-day-recall PROMIS-PA scores with the YAP scores in our study was higher than the association with the step counts. This was expected because step counts measure actual step/movement and objectively capture the volume of step-related PA [33], which is similar but not the same construct as the PROMIS-PA instrument. For example, a previous study that examined the validity of the YAP showed that the correlation between the YAP and wrist-worn ActiGraph data was 0.38 for performed school PA [17]. This correlation is comparable to the correlations reported in our study between the PROMIS-PA or YAP and the average of daily steps in the total sample. The correlation between the PROMIS-PA and YAP instruments was higher than either of these self-reported instruments with step counts measured by wearable devices. Self-reporting may provide complementary information about activities that cannot be captured by measuring body motion that is recorded as step counts. The average scores of the EMA and EoD surveys, which are assumed to provide more accurate estimates than other self-reporting forms [30], showed moderate to high correlations with the 7-day-recall PROMIS-PA, thus confirming the validity of the 7-day recall instrument version.

Our exploratory analyses in this study also supported the validity of both the EMA and EoD versions of the PROMIS-PA. The EMA and EoD ratings moderately correlated with daily steps based on the single-day analyses. This aligns with other study findings validating the EMA or daily reporting with objective monitoring of PA using wearable devices [23,30,31]. Given the moderate correlation, if quantification of activity level is required (e.g., for metabolic or energy expenditure calculations), wearable devices are often viewed as the better data source. Self-reported physical activity (e.g., using YAP or PROMIS) may be better suited for larger clinical trials, natural history, or epidemiological trials for which wearable devices are not feasible or available. Our prior qualitative study [24] indicated that most interviewed participants (17 out of 18 adolescents) reported a preference to completing the EoD surveys over completing multiple short EMAs throughout the day, as well as over the 7-day recall reporting. The reasons provided were that the EoD reporting would be a more convenient option and easier to adhere to than the EMA; moreover, there was such little variation in their activity across days that one day (EoD reporting) would be a sufficient recall period to summarize their performed PA over the day, and could be more accurate than the 7-day recall. The 7-day analyses showed that both the EoD and the 7-day recall versions summarized the EMA reporting or the average of daily steps comparably. The compliance rates of the EoD recall mode were higher than those of the EMA, as evidenced by the frequent pattern of delayed responses to the EMA that were recorded past the allocated time segments across participants. All of the participants in this study completed their 7-day-recall PROMIS-PA. These findings showed that the EoD and 7-day recall reporting may be preferred over more frequent EMAs in adolescents.

This study has some limitations. The sample of convenience had different proportions in race categories than in the general US population, which may limit its generalizability. We note the importance of extending this study to a more diverse population in future studies. Another limitation to generalizability is that this study was conducted during the first year of the COVID-19 pandemic. Although 44% of the participants reported doing less PA during the pandemic, 38% of the participants reported doing more PA during the pandemic, which still provided an extended range of actual PA levels in this study that assessed convergent validity. We also note that due to the increase in home-based schooling during COVID-19, compliance with the EMA may have been enhanced, as children could respond more easily during the day than if they were in school. Even so, 28% of the EMA and 12.57% of the EoD responses were delayed by participants, indicating that many chose to answer at the end of day, whether they were at home or not. Some of the reported reasons for delayed responses included restrictions by parents for electronics time, or limited access when using parents’ devices for younger participants, and being busy with schoolwork/exams. Our study design did not allow for analyses of the differential impact that the dual-reporting modes may have introduced, as all of the participants participated in the EMA, EoD, and 7-day recall modes. We note that the EMA may serve as a reminder to be physically active, and therefore could increase the level of PA; this should be considered in studies using PA level as the endpoint. Finally, although the exploratory part of this study provided some important information about the feasibility of administering the PROMIS-PA’s EMA and EoD versions in adolescents, future studies examining the convergent validity of these recall mode versions need to be investigated in a larger sample size.

## 5. Conclusions

The 7-day-recall PROMIS-PA demonstrated convergent validity with other PA outcome measures (the YAP and wearable devices) and other reporting forms using the PROMIS-PA items (EoD recall and EMA). The effect of the three recall modes, which are the 7-day recall, EoD recall, and the EMA of PROMIS-PA, is comparable. The EoD and EMA versions can be used in pediatric clinical practice or research, when aiming to track lived experiences of PA on a daily basis or throughout the day, respectively. More complete self-reported data about the daily performed PA can be obtained using the EoD alone or in combination with the EMA, than just using the EMA. The 7-day recall and EoD recall PROMIS-PA may be a more practical and sufficient representation of PA in some circumstances when compared to the EMA, as adolescents demonstrated higher compliance rates to these versions.

## Figures and Tables

**Table 1:** Participants’ descriptive characteristics in the total sample (*n* = 84) and sub-sample (*n* = 25).

Variable	Total Sample Summary Statistic (*n* = 84) *n* (%)	Sub-Sample Summary Statistic (*n* = 25) *n* (%)
Gender		
Female	44 (52.4%)	12 (48%)
Male	40 (47.6%)	13 (52%)
Participant race		
White	66 (78.6%)	18 (72%)
African American	2 (2.4%)	-
Asian	11 (13.1%)	7 (28%)
Other	5 (6%)	-
Mother’s race		
White	67 (79.8%)	18 (72%)
African American	2 (2.4%)	-
Asian	11 (13.1%)	6 (24%)
Native Hawaiian and the Pacific Islander	1 (1.2%)	1 (4%)
Other	3 (3.6%)	-
Father’s race		
White	67 (79.8%)	19 (76%)
African American	3 (3.6%)	-
Asian	10 (11.9%)	6 (24%)
Other	4 (4.8%)	-
	**Mean ± SD (range)**	**Mean ± SD (range)**
Age, y	13.99 ± 2.44 (10–20)	14.16 ± 2.09 (11–18)
Weight, kg	57.98 ± 2.44 (23.59–120.20)	65.22 ± 23.12 (28.58–120.20)
Height, cm	162.71 ± 2.44 (137.16–193.04)	166.71 ± 13.57 (142.24–193.04)
The 7-day-recall PROMIS-PA scores	27.70 ± 7.68 (9–40)	29.12 ± 7.94 (12–40)
YAP scores	14.46 ± 6.83 (0–32)	15.52 ± 6.39 (4–32)
Average of daily steps	7936.76 ± 2972.39 (1795.71–17,257.00)	8468.68 ± 3365.68 (3953.14–14,893.43)

Abbreviations: PROMIS-PA, Patient-Reported Outcome Measurement Information System for Pediatric Physical Activity; YAP, Youth Activity Profile; n, number of participants; SD, standard deviation.

**Table 2:** PROMIS-PA reporting versions and scores intended to be collected from participants for associations testing.

PROMIS	DAY							
	1	2	3	4	5	6	7	Average/ Week
**EMA**	m_1_	m_6_	m_11_	m_16_	m_21_	m_26_	m_31_	${\overset{-}{x}}_{m1-m35}$
	m_2_	m_7_	m_12_	m_17_	m_22_	m_27_	m_32_	
	m_3_	m_8_	m_13_	m_18_	m_23_	m_28_	m_33_	
	m_4_	m_9_	m_14_	m_19_	m_24_	m_29_	m_34_	
	m_5_	m_10_	m_15_	m_20_	m_25_	m_30_	m_35_	
**Average EMA/day**	${\overset{-}{x}}_{m1-m5}$	${\overset{-}{x}}_{m6-m10}$	${\overset{-}{x}}_{11-m15}$	${\overset{-}{x}}_{m16-m20}$	${\overset{-}{x}}_{m21-m25}$	${\overset{-}{x}}_{m26-m30}$	${\overset{-}{x}}_{m31-m35}$	
**EoD**	d_1_	d_2_	d_3_	d_4_	d_5_	d_6_	d_7_	${\overset{-}{x}}_{d1-d7}$
**7-days recall**							w	

Note: This table describes all the PROMIS-PA scores that researchers aimed to collect from participants using the 7-day recall, EMA, and EoD forms, as well as the days in which they had to be recorded. A total of 5 EMA scores and an EoD score had to be collected from each participant every day during the scheduled days, and a 7-day-recall PROMIS-PA score had to be collected on the 7th day. The 5 EMA scores for each day had to be averaged (“Average EMA/day” in the table) for inclusion in the single-day analysis. The total 35 EMA scores from the entire week, as well as the EoD scores from all the days of the week, had to be averaged (“Average/week” in the table) for inclusion in the 7-day analysis. Only the 7-day recall PROMIS-PA data from this table were included in the total sample analyses. Abbreviations: PROMIS-PA, Patient-Reported Outcome Measurement Information System for Pediatric Physical Activity; EMA, Ecological Momentary Assessment; EoD, End of Day; “m_i_” is the EMA version scores where “i” is the number of a prompt; “d_k_” is the end-of-day version scores for each k day; “w” is the 7-day recall version score at the end of the week (on the 7th day); “$\overset{-}{x}$ ” is the mean of weekly or daily scores; average EMA/day = ${\overset{-}{x}}_{m\sum i\;for\;each\;day}$.

**Table 3:** Numbers of participants with valid EMA and EoD data for single-day analyses in the sub-sample (*n* = 25).

Day of Activity Tracking	Number of Participants Completed 3–5 EMA Reports within the Allocated Time Segments	Number of Participants Completed EoD Reports before 2 a.m.
1	22 (88%)	22 (88%)
2	22 (88%)	20 (80%)
3	19 (76%)	24 (96%)
4	16 (64%)	24 (96%)
5	16 (64%)	21 (84%)
6	15 (60%)	19 (76%)
7	17 (68%)	22 (88%)

Abbreviations: EMA, Ecological Momentary Assessment; EoD, end of day; *n*, number of participants.

**Table 4:** Pearson’s correlations (r) from the 7-day analyses run in the sub-sample (*n* = 25).

Variable	1	2	3	4
1. Average EMA $\left({\overset{-}{x}}_{m1-m35}\right)$	_			
2. Average EoD $\left({\overset{-}{x}}_{d1-d7}\right)$	0.788 ** n = 20	_		
3. 7-day-recall PROMIS-PA	0.748 ** n = 21	0.914 ** n = 24	_	
4. Average of daily steps	0.505 * n = 18	0.489 * n = 21	0.569 ** n = 22	_

Note: This table describes the associations between the average of all EMA scores over the week (1), average of all EoD scores over the week (2), 7-day-recall PROMIS-PA score on the 7th day (3), and average of daily steps over the week (4). Abbreviations: PROMIS-PA, Patient-Reported Outcome Measurement Information System for Pediatric Physical Activity; EMA, Ecological Momentary Assessment; EoD, end of day; $\left({\overset{-}{x}}_{m1-m35}\right)$, the mean of all valid EMA scores across the week; $\left({\overset{-}{x}}_{d1-d7}\right)$, the mean of all valid EoD scores across the week; *n,* number of participants involved in each correlation based on available valid data from both variables * *p* < 0.05. ** *p* < 0.01.

**Table 5:** Pearson’s correlations (r) from the single-day analyses run in the sub-sample (*n* = 25).

	Day 1	Day2	Day 3	Day 4	Day 5	Day 6	Day 7
Correlation between	r = 0.836	r = 0.638	r = 0.860	r = 0.813	r = 0.784	r = 0.772	r = 0.945
average daily EMA ratings and	*p* < 0.001	*p* = 0.004	*p* < 0.001	*p* < 0.001	*p* = 0.001	*p* = 0.001	*p* < 0.001
EoD score	*n* = 19	*n* = 18	*n* = 19	*n* = 16	*n* = 14	*n* = 14	*n* = 16
Correlation between	r = 0.542	r = 0.561	r = 0.581	r = 0.563	r = 0.439	r = 0.669	r = 0.675
average daily EMA ratings and	*p* = 0.014	*p* = 0.010	*p* = 0.018	*p* = 0.036	*p* = 0.133	*p* = 0.012	*p* = 0.006
daily steps	*n =* 20	*n =* 20	*n =* 16	*n =* 14	*n =* 13	*n =* 13	*n =* 15
Correlation between	r = 0.465	r = 0.283	r = 0.543	r = 0.493	r = 0.612	r = 0.513	r = 0.661
EoD score and	*p* = 0.020	*p* = 0.272	*p* = 0.011	*p* = 0.023	*p* = 0.007	*p* = 0.025	*p* = 0.002
daily steps	*n =* 19	*n =* 17	*n =* 21	*n =* 21	*n =* 18	*n =* 19	*n =* 19

Abbreviations: EMA, Ecological Momentary Assessment; EoD, end of day; r, Pearson’s correlation coefficient; *n*, number of participants.

## Data Availability

The datasets used and/or analyzed during the current study are available from the corresponding author upon reasonable request.

## Appendix A

PROMIS Pediatric Physical Activity (Eight Questions)

Please respond to each question or statement by choosing one answer

1. In the past 7 days, how many days did you exercise or play so hard that your body got tired?

(1) No days (2) 1 day (3) 2–3 days (4) 4–5 days (5) 6–7 days

2. In the past 7 days, how many days did you exercise really hard for 10 min or more?

(1) No days (2) 1 day (3) 2–3 days (4) 4–5 days (5) 6–7 days

3. In the past 7 days, how many days did you exercise so much that you breathed hard?

(1) No days (2) 1 day (3) 2–3 days (4) 4–5 days (5) 6–7 days

4. In the past 7 days, how many days were you so physically active that you sweated?

(1) No days (2) 1 day (3) 2–3 days (4) 4–5 days (5) 6–7 days

5. In the past 7 days, how many days did you exercise or play so hard that your muscles burned?

(1) No days (2) 1 day (3) 2–3 days (4) 4–5 days (5) 6–7 days

6. In the past 7 days, how many days did you exercise or play so hard that you felt tired?

(1) No days (2) 1 day (3) 2–3 days (4) 4–5 days (5) 6–7 days

7. In the past 7 days, how many days were you physically active for 10 min or more?

(1) No days (2) 1 day (3) 2–3 days (4) 4–5 days (5) 6–7 days

8. In the past 7 days, how many days did you run for 10 min or more?

(1) No days (2) 1 day (3) 2–3 days (4) 4–5 days (5) 6–7 days

## Appendix B

Ecological Momentary Assessment and End-of-Day Versions of the PROMIS-PA

### Appendix B.1. The PROMIS-PA Ecological Momentary Assessment Version (Four Questions—The Early Morning Survey as an Example)

Please respond to each question by choosing one answer *

1. From about 6 to 8 a.m., did you exercise or play so hard that your body got tired?

☐Yes  ☐No

2. From about 6 to 8 a.m., did you exercise really hard for 10 min or more?

☐Yes  ☐No

3. From about 6 to 8 a.m., did you exercise so much that you breathed hard?

☐Yes  ☐No

4. From about 6 to 8 a.m., were you being so physically active that you sweated?

☐Yes  ☐No

### Appendix B.2. The PROMIS-PA End of Day Recall Version (Eight Questions)

Please respond to each question by choosing one answer *

1. Today, did you exercise or play so hard that your body got tired?

☐Yes  ☐No

2. Today, did you exercise really hard for 10 min or more?

☐Yes  ☐No

3. Today, did you exercise so much that you breathed hard?

☐Yes  ☐No

4. Today, were you so physically active that you sweated?

☐Yes  ☐No

5. Today, did you exercise or play so hard that your muscles burned?

☐Yes  ☐No

6. Today, did you exercise or play so hard that you felt tired?

☐Yes  ☐No

7. Today, were you physically active for 10 min or more?

☐Yes  ☐No

8. Today, did you run for 10 min or more?

☐Yes  ☐No

* The “Yes” and “No” responses were scored as 1 and 0 values, respectively, to calculate the summed score for each EAM or EoD survey.

## Appendix C

**Table A1:** Time frames as reported in the EMA and EoD survey items for each time segment during the day, and when they are considered valid responses for subsequent analysis based on the completion time by participants.

Time Segment	Survey Items Asking about PA Performed during This Time Segment	Coded as Completed within Allocated Time Segment If the Survey Was Completed at:
EM	“From about 6 to 8 a.m.,…”	8 a.m. to 12 p.m.
MLM	“From about 8 a.m. to 12 p.m.,…”	12 to 3 p.m.
EA	“From about 12 to 3 p.m.,…”	3 to 6 p.m.
LA	“From about 3 to 6 p.m.,…”	6 to 8 p.m.
E	“From about 6 p.m. until your bedtime,…”	8 p.m. to 2 a.m.
**EoD**	“Today,…” (available at 8 p.m.)	8 p.m. to 2 a.m.

Abbreviations: PA, physical activity; EM, early morning; MLM, mid-to-late morning; EA, early afternoon; LA, late afternoon; E, evening; EoD, end-of-day PROMIS-PA version.

## Appendix D

Modified Youth Activity Profile (YAP)

These questions ask about your overall levels of physical activity during different periods of time. This would include structured exercise or sport activities as well as activity playing with friends, dancing or doing work/chores. Answer the questions based on your physical activity in the last 7 days

1. Activity Early Morning: How many days in the early morning (6:00–8:00 a.m.) did you do some form of physical activity for at least 10 min?

a. 0 days b. 1 day c. 2 days d. 3 days e. 4–5 days f. 6–7 days

2. Activity Mid-Late Morning: How many days in the mid-late morning (8:00 a.m.–12:00 p.m.) did you do some form of physical activity for at least 10 min?

a. 0 days b. 1 day c. 2 days d. 3 days e. 4–5 days f. 6–7 days

3. Activity Early Afternoon: How many days in the early afternoon (12:00–3:00 p.m.) did you do some form of physical activity for at least 10 min?

a. 0 days b. 1 day c. 2 days d. 3 days e. 4–5 days f. 6–7 days

4. Activity Late Afternoon: How many days in the late afternoon (between 3:00–6:00 p.m.) did you do some form of physical activity for at least 10 min? (This can include playing with your friends/family, team practices or classes involving physical activity).

a. 0 days b. 1 day c. 2 days d. 3 days e. 4–5 days f. 6–7 days

5. Activity on Evenings: How many evenings (6:00–10:00 p.m.) did you do some form of physical activity for at least 10 min? (This can include playing with your friends/family, team practices or classes involving physical activity).

a. 0 days b. 1 day c. 2 days d. 3 days e. 4–5 days f. 6–7 days

6. Activity on Saturday: How much physical activity did you do last Saturday? (This could be for exercise, work/chores, family outings, sports, dance, or play. If you don’t remember, try to estimate)

a. No activity (0 min) b. Small amount of activity (1 to 30 min) c. Small to Moderate amount of activity (31 to 60 min) d. Moderate to Large amount of activity (1 to 2 h) e. Large amount of activity (more than 2 h)

7. Activity on Sunday: How much physical activity did you do last Sunday? (This could be for exercise, work/chores, family outings, sports, dance, or play. If you don’t remember, try to estimate)

a. No activity (0 min) b. Small amount of activity (1 to 30 min) c. Small to Moderate amount of activity (31 to 60 min) d. Moderate to Large amount of activity (1 to 2 h) e. Large amount of activity (more than 2 h)
